# The Comparation of Arrhenius-Type and Modified Johnson–Cook Constitutive Models at Elevated Temperature for Annealed TA31 Titanium Alloy

**DOI:** 10.3390/ma16010280

**Published:** 2022-12-28

**Authors:** Shengli Yang, Pei Liang, Fuyang Gao, Dejun Song, Peng Jiang, Min Zhao, Ning Kong

**Affiliations:** 1 Luoyang Ship Material Research Institute, Luoyang 471039, China; 2 School of Mechanical Engineering, University of Science and Technology Beijing, Beijing 100083, China; 3 Skate Key Laboratory of Ocean Engineering, School of Naval Architecture, Ocean and Civil Engineering, Shanghai Jiao Tong University, Shanghai 200240, China; 4 State Key Laboratory of High Performance Complex Manufacturing, Central South University, Changsha 410083, China

**Keywords:** titanium alloy, constitutive model, Arrhenius model, Johnson–Cook model, softening phenomenon

## Abstract

Constitutive models play a significant role in understanding the deformation behavior of materials and in optimizing the manufacturing process. In order to improve the reliability of calculation results, the high temperature flow behavior of TA31 titanium alloy obtained from an annealed hot-rolled plate has been investigated by a Gleeble-3500 thermo-mechanical simulator. The isothermal hot compression tests are conducted in the temperature range of 850 to 1050 °C and the strain rate range from 0.001 to 10 s^−1^ with a height reduction of 60%. The annealed TA31 shows a dynamic recovery characteristic during thermo-mechanical processing. The experimental data have been used to develop an Arrhenius-type constitutive model and a modified Johnson–Cook model under the consideration of coupling effect on strain, temperature, and strain rate, as well as the strain-softening phenomenon. The material parameters are determined by a global optimization method based on the initial values by means of a regression method. A comparation of the predicted results has been performed based on the modified Johnson–Cook model and those acquired from the Arrhenius-type model. The correlation coefficient and average absolute relative error of the modified Johnson–Cook model are 4.57% and 0.9945, respectively. However, when the optimization method has been applied, they are 15.77% and 0.9620 for the Arrhenius-type model, respectively. These results indicate that the modified Johnson–Cook model is more accurate and efficient in predicting the flow stress of annealed TA31 titanium alloy under a set of model material parameters. Furthermore, the simple mathematical expression of this model is helpful to incorporate it into the finite element software to obtain detailed and valuable information during the thermo-mechanical processing simulation for TA31 in further work.

## 1. Introduction

Owing to its excellent properties, such as a high strength-to-weight ratio and strong corrosion resistance [1], TA31 titanium alloy is considered as an attractive material for certain parts of ships. It is well known that thermo-mechanical processing (TMP) is a highly non-linear dynamic process [2,3,4,5], including both elastic deformation and plastic deformation of the workpiece, and the accompanying high temperature, high strain rate, as well as complex and variable friction conditions. Constitutive models always are used to describe the relationship between the dynamic material response (stress) and processing parameters (strain, strain rate, and temperature). An appropriate constitutive model should simultaneously consider the coupled effects of deformation temperature, strain, and strain rate. It is also necessary to capture the flow behavior of materials accurately. This is significant for understanding the deformation behavior and the optimization of the deformation process of the alloy [6,7].

There are three categories of constitutive models [8,9,10]: empirical models, semi-empirical models, and models incorporating physical phenomena. The last two classes are used primarily to reflect the evolution of specific physics during TMP, such as recrystallization, grain size, twinning, and so on, which lead to the complexity of usedness. The empirical models generally have a simple formulation, which can be undertaken at low computational and experimental costs. It could be facilitated to implement into numerical software. The Johnson–Cook (JC) [11,12,13,14] and Arrhenius-type models [15,16,17] are the most commonly used ones for various alloys. However, the original JC model shows good prediction only close to the reference strain rate and reference temperature [11]. It has no adequate ability to predict the flow stress of titanium alloys, especially in wide temperature ranges including dual phase and single phase [12,13]. Many efforts have been made to improve the predictability of the original JC model, while retaining the low number of material parameters and identifying them easily [18,19]. The Arrhenius-type model also has undergone developments in recent years due to its simple form and the ability to reflect the activation energy related to the microstructure evolution [20].

It is well known that no constitutive model is perfect and can be applied to all complex conditions and all materials. Therefore, many comparative studies on constitutive relationships for different materials have been conducted. Peng et al. [7] compared the predictability of the Arrhenius-type and artificial neural network models for the as-cast Ti60 titanium alloy. He et al. [21] established the JC, modified JC, and Arrhenius-type constitutive models for a 20CrMo alloy steel. The accuracy and effectiveness of the three models are comparatively studied subsequently. Chen et al. [22] proposed a segmented model for AZ80 magnesium alloy based on the modified Arrhenius-type relation and JC model with high prediction accuracy. Although the above models have been established and compared, there is still a lack of practical and accurate models to calculate the flow behavior of annealed TA31. It is essential to seek a suitable constitutive model to exhibit high accuracy, which is easy to combine with FEM [23,24].

In this work, a comparative study on the constitutive relationships of annealed TA31 titanium alloy based on Arrhenius-type and modified Johnson–Cook models at high temperatures has been completed. The flow behavior of annealed TA31 titanium alloy in temperature ranges of 850 to 1050 °C and strain rates ranges of 0.001 to 10 s^−1^ is investigated by a Gleeble-3500 thermo-mechanical simulator. The Arrhenius-type constitutive model considering strain compensation and the modified Johnson–Cook model incorporating the interaction of strain, strain rate, and temperature are established and compared. This is helpful to improve the systematic study on the annealed TA31 and provide theoretical guidance for optimizing the process parameters.

## 2. Experiments and Results

### 2.1. Experimental Procedures

**Figure 1:**
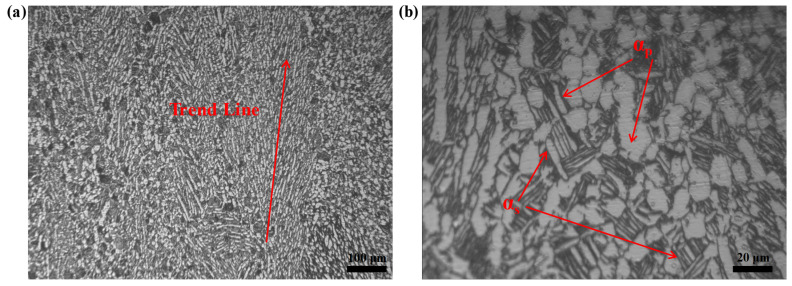
Initial microstructure of TA31 in the as-received condition: (**a**) 100×; (**b**) 500×.

The material used in this experiment is the titanium alloy TA31, and its nominal chemical composition (wt.%) is Ti-6Al-3Nb-2Zr-1Mo, as shown in Table 1. Titanium is characterized by allotropic behavior including the close-packed (HCP) crystal structure (α), and body-centered cubic (BCC) crystal structure (β), which are related to the temperature [1]. The α phases initially presented in the material and retained at deformation temperature usually globularize and appear as equiaxed α grains after cooling. They are known as the primary α phases (α_p_). On the other hand, the α phases from the decomposition of the hot-worked β phases during cooling show lamella with a colony morphology. They are called secondary α phases (α_s_) [25]. The starting microstructure is shown in Figure 1. The initial microstructure of annealed TA31 is a typical bimodal structure [26]. It is composed of equiaxed α_p_ and lamellar α_s_ in the continuous β matrix (black region). In addition, it is observed that some α phases show a local tendency (Figure 1a).

**Figure 2:**
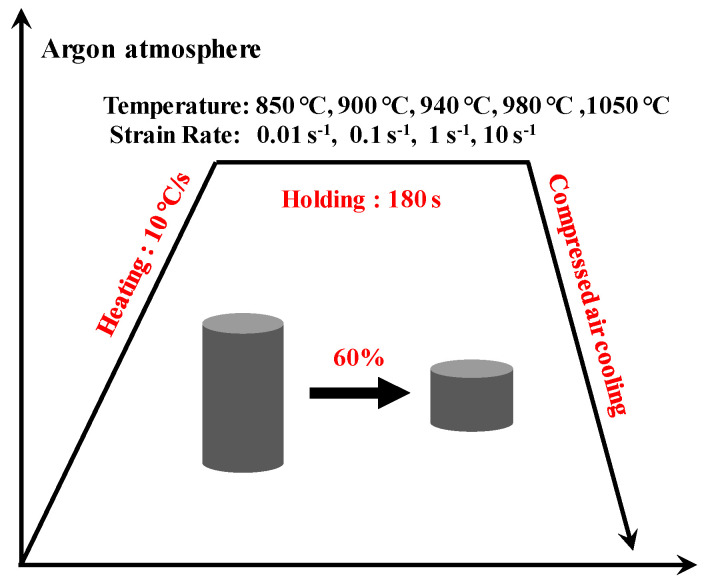
Schematic illustration of the isothermal compression process.

The specimens for isothermal hot compression tests are machined into a cylinder with 10 mm in diameter and 15 mm in height. These tests are conducted on a Gleeble-3500 thermo-mechanical simulator. The β-transus temperature of this alloy is 1000 ± 7 °C. The deformation temperatures are 850, 900, 940, 980, and 1050 °C. The strain rates are 0.01, 0.1, 1.0, and 10 s^−1^. The schematic illustration of the isothermal compression process is shown in Figure 2. All specimens are heated to the targeted temperature and held for 3 min to obtain a uniform temperature distribution. The height reduction of the compression tests is 60%, and argon protection is used to avoid the effect of air. The true stress–strain curves are recorded automatically in the hot compression process.

### 2.2. Analysis of Flow Stress Behavior

**Figure 3:**
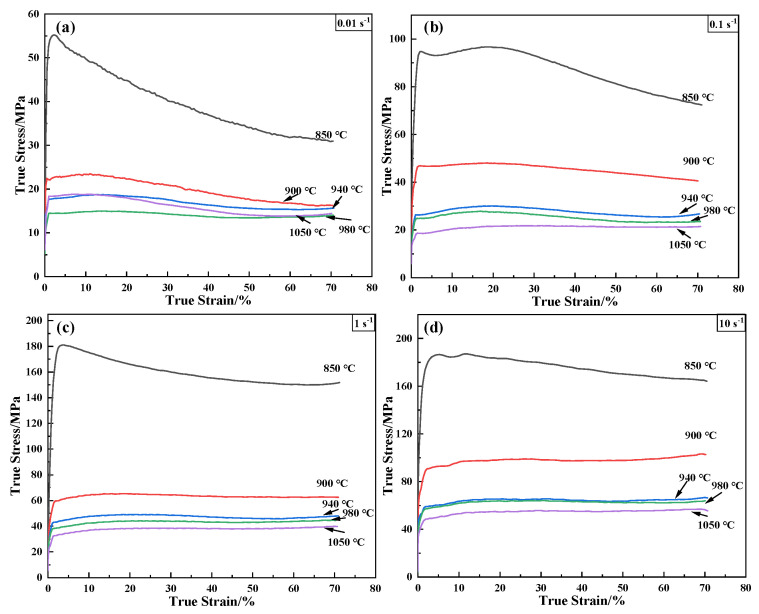
True stress–strain curves of annealed TA31 alloy under different strain rates: (**a**) 0.01 s^−1^; (**b**) 0.1 s^−1^; (**c**) 1 s^−1^; and (**d**) 10 s^−1^.

Figure 3 shows the flow curves of the annealed TA31 in the α + β two-phase region and β single-phase region at different deformation conditions. The shape of the stress–strain curves is the competitive result of work hardening and dynamic softening. Specifically, the flow stress abruptly increases to a peak and then decreases until obtaining a steady state when the temperature is 850 °C and the strain rate is in the range of 0.01~10 s^−1^. It is the equilibrium of work hardening and dynamic recovery/recrystallization [28]. Higher temperatures and lower strain rates supply a longer time for energy accumulation and higher mobility at boundaries for the nucleation and growth of dynamically recrystallized grains and dislocation annihilation [29,30]. As a result, the stress–strain curves do not show an apparent hardening phenomenon at other higher temperatures and all strain rates because the work softening is predominant. The TA31 alloy is a negative temperature-sensitive and a positive strain rate-sensitive material. The stress will gradually increase with the rising strain rates under a constant deformation temperature. It will decrease with increasing deformation temperatures at a constant deformation strain rate. However, the variation amplitude is different in the same temperature or strain rate increment. It is concluded that the flow stress of the TA31 alloy is sensitive to the deformation strain rate and temperature. Establishing a suitable constitutive model is essential for the simulations and subsequent processing of annealed TA31.

## 3. Establishment of the Constitutive Relationship of Annealed TA31

### 3.1. The Arrhenius-Type Model

The Arrhenius-type model is the most popular constitutive model for the hot forming of metals. There is an interactive relationship between the flow stress, strain rate, and temperature at a certain strain during hot-working deformation [31]. The Zener–Hollomon parameter *Z* is introduced and is responsible for showing the dependence of temperature and strain rate on the thermal deformation behavior of metals and alloys.

(1) $$Z=\dot{\varepsilon }\exp\left(Q/RT\right)$$

where $\dot{\varepsilon }$ is the strain rate(s^−1^), *Q* refers to the activation energy for hot deformation (kJ/mol), *R* represents the universal gas constant (8.3145 J mol^−1^ K^−1^), and *T* is the workpiece temperature (K). The flow stress σ (MPa) can be written in an explicit form with a newly introduced material parameter *A* as follows.

(2) $$\sigma =\frac{1}{\alpha }\ln\left\{{\left(\frac{Z}{A}\right)}^{1/n}+{\left[{\left(\frac{Z}{A}\right)}^{2/n}+1\right]}^{1/2}\right\}$$

Plastic strain significantly affects flow stress due to microstructure evolution during the deformation, such as strain hardening and dynamic softening. Hence, the compensations of strain are considered by establishing polynomials of material constants and strain (*α*, *n*, *Q*, and ln*A*) to obtain a more precise and accurate prediction for the flow behavior of materials. The 5th-order polynomials are chosen to represent the influence of strain on material constants.

(3) $$\alpha \left(\varepsilon \right)=\alpha_{0}+\alpha_{1}\varepsilon +\alpha_{2}\varepsilon^{2}+\alpha_{3}\varepsilon^{3}+\alpha_{4}\varepsilon^{4}+\ldots +\alpha_{m}\varepsilon^{m}$$

(4) $$n\left(\varepsilon \right)=n_{0}+n_{1}\varepsilon +n_{2}\varepsilon^{2}+n_{3}\varepsilon^{3}+n_{4}\varepsilon^{4}+\ldots +n_{m}\varepsilon^{m}$$

(5) $$lnA\left(\varepsilon \right)=lnA_{0}+lnA_{1}\varepsilon +lnA_{2}\varepsilon^{2}+lnA_{3}\varepsilon^{3}+lnA_{4}\varepsilon^{4}+\ldots +lnA_{m}\varepsilon^{m}$$

(6) A(ε)=exp[lnA(ε)]

(7) $$Q\left(\varepsilon \right)=Q_{0}+Q_{1}\varepsilon +Q_{2}\varepsilon^{2}+Q_{3}\varepsilon^{3}+Q_{4}\varepsilon^{4}+\ldots +Q_{m}\varepsilon^{m}$$

The order *m* of the polynomial is varied from one to nine. An optimal polynomial order is selected based on the analysis of correlation and generalization.

### 3.2. The Modified Johnson–Cook Model

Besides the Arrhenius-type model, the JC model [32] is also often applied to the forming process due to its few parameters, limited experiments needed, and low fitted complexity.

(8) $$\sigma =\left(A+B\cdot \varepsilon^{n}\right)\cdot \left(1+C\cdot \ln{\dot{\varepsilon }}^{*}\right)\cdot \left(1-T^{*}{}^{m}\right)$$

(9) $$T^{*}=\frac{T-T_{r}}{T_{m}-T_{r}}$$

(10) $${\dot{\varepsilon }}^{*}=\frac{\dot{\varepsilon }}{{\dot{\varepsilon }}_{0}}$$

where *A* (Mpa) is the yield stress at the reference temperature *T_r_* (°C) and equivalent plastic ${\dot{\varepsilon }}_{0}$(*s*^−1^), *B* is the factor of strain, *n* is the strain-hardening exponent, *C* represents the coefficient of strain rate hardening, and *m* is the thermal-softening exponent. However, the original model cannot capture the flow behavior of some materials, particularly for the titanium alloys with temperature and strain rate sensitivity. Liqun et al. [33] proposed a modified JC constitutive model considering the coupling effect of strain, temperature, and strain rate, as well as the strain-softening phenomenon.

(11) $$\sigma =\left(K_{1}\varepsilon^{K_{2}+K_{3}\varepsilon +K_{4}/\varepsilon }\right)\left(1+E\ln{\dot{\varepsilon }}^{*}\right)\exp\left(FT^{*}\right)$$

(12) $$E=E_{0}+E_{1}\varepsilon +E_{2}\varepsilon^{2}+E_{3}\dot{\varepsilon }+E_{4}{\dot{\varepsilon }}^{2}+E_{5}\varepsilon \dot{\varepsilon }$$

(13) $$F=F_{0}+F_{1}\varepsilon +F_{2}\dot{\varepsilon }+F_{3}T^{*}+F_{4}\varepsilon^{3}+F_{5}{\dot{\varepsilon }}^{3}+F_{6}T^{*}{}^{3}+F_{7}\varepsilon \dot{\varepsilon }T^{*}$$

where the *K*_1_, *K*_2_, *K*_3_, and *K*_4_ are the material coefficients of the strain term, which reflects the strain-softening mechanism. The parameter *E* is a function of ε and $\dot{\varepsilon }$. The parameter *F* is also a function of the mutual coupling of ε, $\dot{\varepsilon }$, and *T*.

### 3.3. The Calibration of Material Parameters

**Figure 4:**
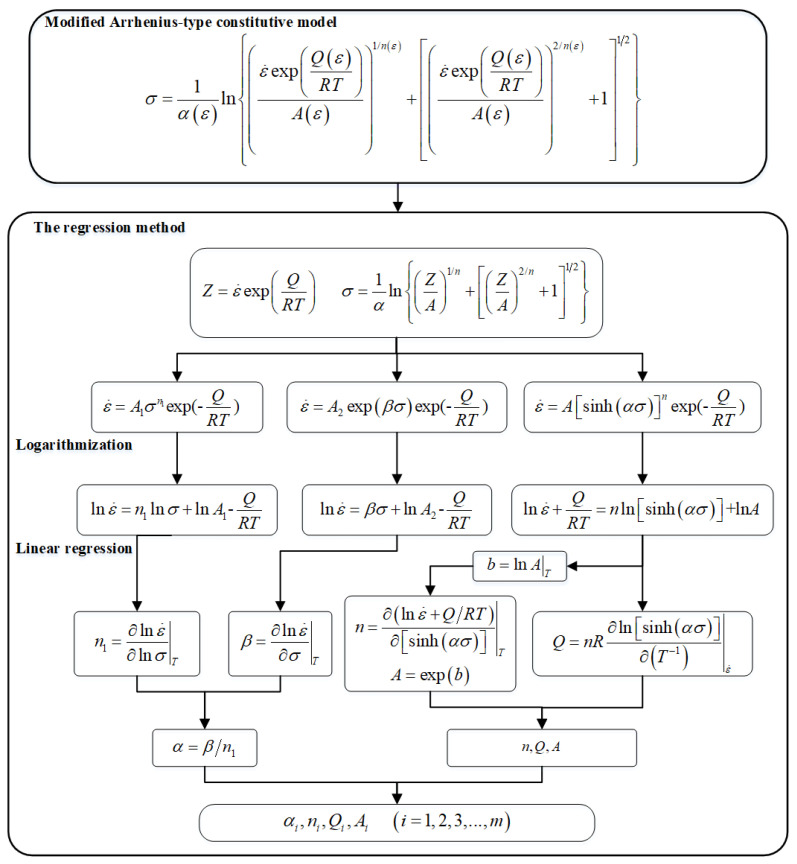
Flowchart of material constants’ calibration implementation by regression method.

**Figure 5:**
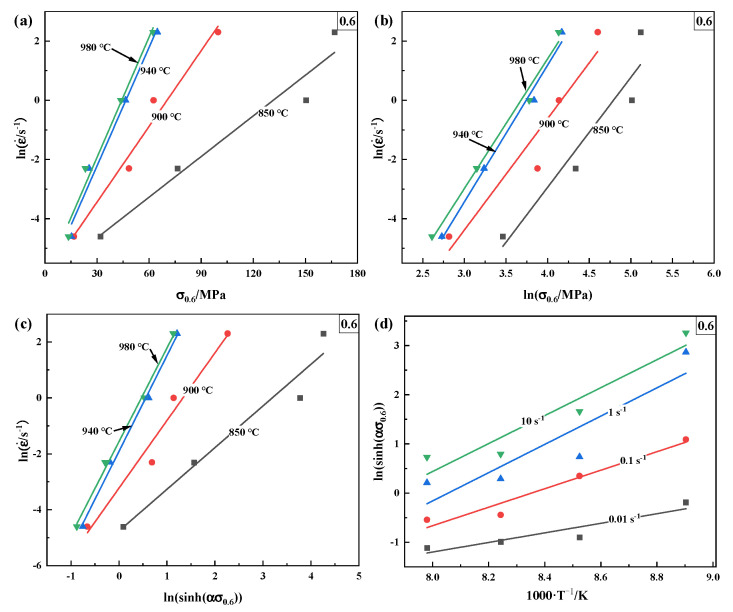
Relationships between the following: (**a**) $\ln\dot{\varepsilon }-\sigma$; (**b**) $\ln\dot{\varepsilon }-\ln\sigma$; (**c**) $\ln\dot{\varepsilon }-\ln[\sinh(\alpha \sigma )]$; (**d**) $\ln[\sinh(\alpha \sigma )]-1000\cdot T^{-1}$.

**Figure 6:**
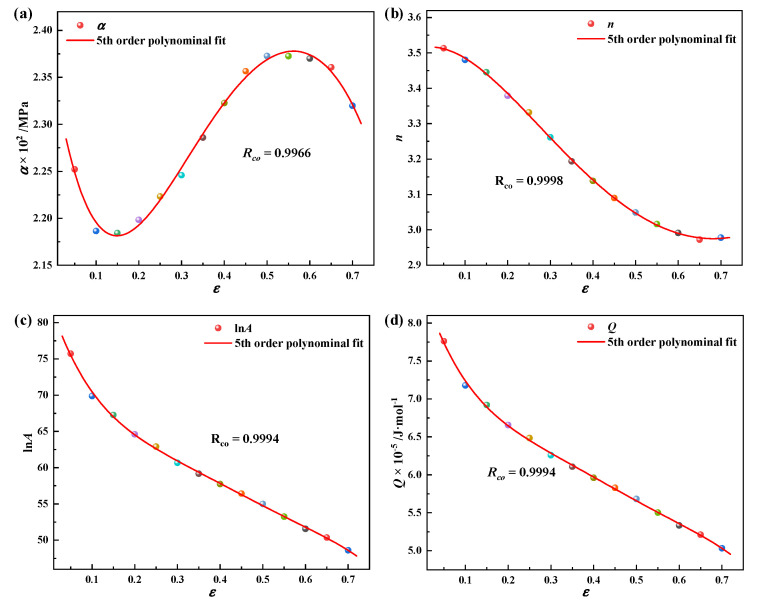
Variation of (**a**) *α*, (**b**) *n*, (**c**) ln*A*, and (**d**) *Q* with true strain using 5th polynomial fit.

As shown in Figure 4, the material constants in the Arrhenius-type model are obtained by the equation logarithmization and liner fitting with some specific data points under the same strains for each stress–strain flow curve. In addition, many papers have described the calibration of material constants in detail [21,34]. As shown in Figure 5, the average slopes and the intercept of the liner fitting of $\ln\dot{\varepsilon }$−σ, $\ln\dot{\varepsilon }$−lnσ, $\ln\dot{\varepsilon }$−ln[sinh(ασ)], and ln[sinh(ασ)]−1000·T^−1^ at a particular true strain of 0.6 are obtained, respectively. Then, the value of α, *n*, ln*A*, and *Q* can be gained by the calculation scheme as depicted in Figure 4. Those values within the strain range of 0.05–0.7 within an interval of 0.05 are repeatedly calculated by the same method. The 5th-order polynomials represent the relationships between these material parameters and plastic strain with a good correlation and generation, as described in Figure 6. The corresponding values of these polynomial constants are listed in Table 2. For the modified JC model, the coefficients are also determined by the regression analysis detailed in Liqun’s research [33].

Unfortunately, the coefficient calibrations of the above models are tedious and time-consuming. Furthermore, only a few experimental data are used, which may lead to significant prediction errors. A new calibration method has been proposed with the help of the development of optimization algorithms, and has been explained in detail in our previous research [35]. The optimization objective is to minimize the area between the experiment flow curve and its corresponding predicted curve at the current step. The coefficients in Table 2 and Table 3 are set as initial values for the optimization procedure, and Table 4 and Table 5 present a series of optimized results.

## 4. Comparison of Both Models

Both constitutive models in the temperature range of 850–980 °C and strain rate range of 0.01–10 s^−1^ have been developed after determining all material constants. The correlation coefficient (*R_co_*) and average absolute relative error (*AARE*) as standard statistical parameters are used to quantitatively evaluate the reliability and predictability of both the models mentioned above [34,36,37]. *R_co_* could reflect the linear relationship between the experimental and calculated results with accuracy and effectiveness, and *AARE* is a universal statistical parameter for measuring the predictability of a numerical model.

(14) $$R_{co}=\frac{\sum_{i=1}^{N}\left(\sigma_{\exp}^{i}-{\bar{\sigma }}_{\exp}\right)\left(\sigma_{p}^{i}-{\bar{\sigma }}_{p}\right)}{\sqrt{\sum_{i=1}^{N}{\left(\sigma_{\exp}^{i}-{\bar{\sigma }}_{\exp}\right)}^{2}\sum_{i=1}^{N}{\left(\sigma_{p}^{i}-{\bar{\sigma }}_{p}\right)}^{2}}}$$

(15) $$AARE=\frac{1}{N}\sum_{i=1}^{N}\left|\frac{\sigma_{\exp}^{i}-\sigma_{p}^{i}}{\sigma_{\exp}^{i}}\right|\times 100\%$$

where $\sigma_{\exp}^{i}$ is experimental stress, $\sigma_{p}^{i}$ is the predicted stress calculated by the constitutive equation, and ${\bar{\sigma }}_{\exp}$ and ${\bar{\sigma }}_{p}$ are the average values of $\sigma_{\exp}^{i}$ and $\sigma_{p}^{i}$, respectively. *n* is the total number of data employed in the assessment. More than 2000 data points are used to evaluate the predictability of the established constitutive models in this study.

**Figure 7:**
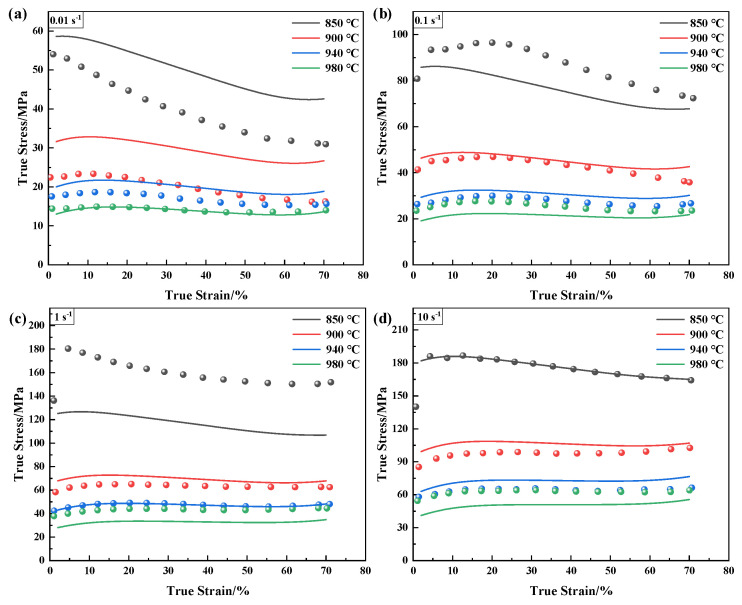
The comparison between the experimental data and predicted data of the Arrhenius-type model at different strain rates: (**a**) 0.01 s^−1^; (**b**) 0.1 s^−1^; (**c**) 1 s^−1^; and (**d**) 10 s^−1^.

**Figure 8:**
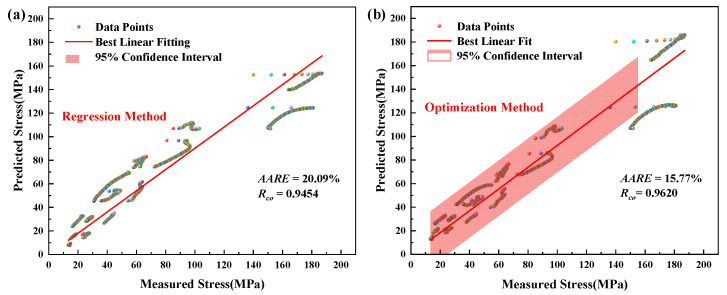
Correlation between measured and predicted stress of the modified Arrhenius-type model: (**a**) regression method; (**b**) optimization method.

As depicted in Figure 7, there are apparent deviations between the experimental data and the predicted data of the Arrhenius-type model, particularly for the high temperature and low strain rate. As can be seen in Figure 8, the *R_co_* and *AARE* are 20.09% and 0.9454 based on the parameters obtained by the regression method, respectively. In addition, they are improved to 15.77% and 0.9620 through the global optimization method, respectively. The Arrhenius-type model cannot accurately represent the flow behavior using only one set of parameters for the annealed TA31, because the temperature range includes the α and α + β ranges with the different deformation mechanisms [38].

**Figure 9:**
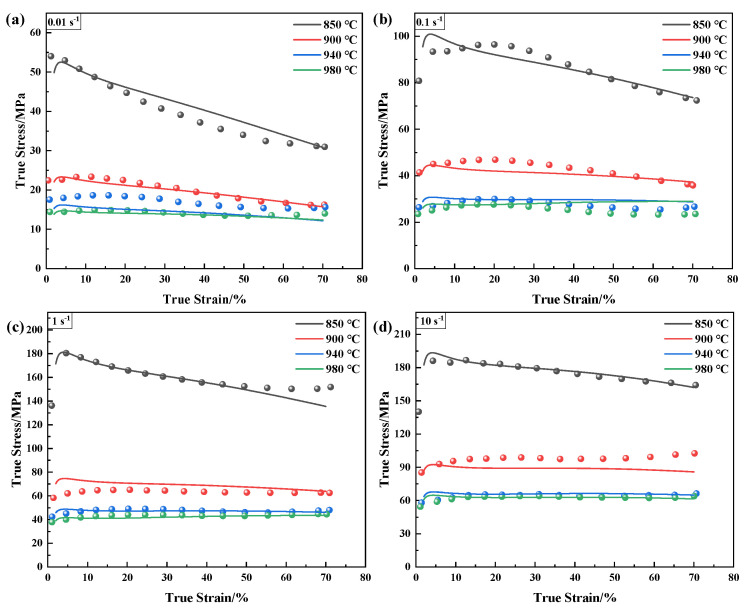
The comparison between the experimental data and predicted data of the modified JC model at different strain rates: (**a**) 0.01 s^−1^; (**b**) 0.1 s^−1^; (**c**) 1 s^−1^; and (**d**) 10 s^−1^.

**Figure 10:**
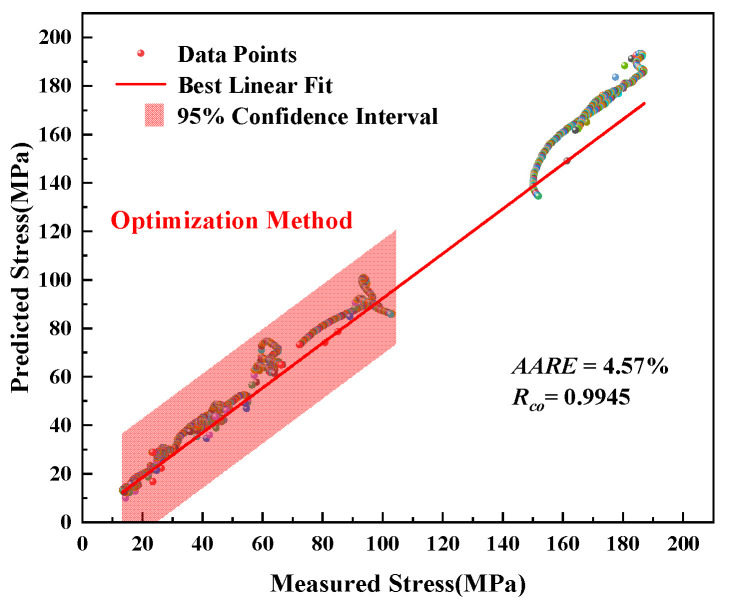
Correlation between measured and predicted stress of the modified JC model by optimization method.

For the modified JC model in Figure 9, the predicted data can efficiently represent the flow behavior of this material under the entire experimental conditions. This describes the characteristics from dynamic recrystallization to dynamic recovery, leading to the change of the curves’ shape. As shown in Figure 10, the red full line indicates the exactly suitable placement between the predicted and experimental values. The Arrhenius-type model has a more significant number of data points deviating from the solid red line (Figure 8). However, almost all the data points of the modified JC model are around the best linear fit. The *R_co_* and *AARE* are 4.57% and 0.9945 based on the parameters obtained by the optimization method, respectively.

The result shows that the modified JC model has better flexibility and predictability than the Arrhenius-type model. This material constitutive model contains the necessary deformation parameters and their interrelationships. It does not need to express piecewise with different rheological parameters to keep the error between the prediction and experiment within a reasonable range [7,22]. The simplicity of the modified JC model and a set of parameters contributes to its implementation in many commercial finite element software.

## 5. Conclusions

In this work, both the strain–stress constitutive models were established based on the experimental data of annealed TA31 in the deformation temperature range of 850–980 °C with the strain rate range of 0.01–10 s^−1^. The accurate constitutive models can significantly improve the performance of the product of the continuous forming process. The concrete results are as follows:

1. The annealed TA31 is a material with negative temperature sensitivity and positive strain rate sensitivity. The dominant deformation mechanism is dynamic recrystallization during low temperatures (˂940 °C), and dynamic recovery during high temperatures (≥940 °C).
2. The Arrhenius-type constitutive model considering strain compensation has been established for TA31. The 5th-order polynomial curves are used to describe the relationships between plastic strain and material parameters. The *R_co_* and *AARE* are 20.09% and 0.9454 based on the parameters obtained by the regression method, respectively. In addition, they are improved to 15.77% and 0.9620 through the global optimization method, respectively.
3. The modified Johnson–Cook model incorporating the coupling effect of strain, temperature, and strain rate, as well as the strain-softening phenomenon is used. The new modified JC model has better correlation and smaller errors. Its *R_co_* and *AARE* are 4.57% and 0.9945 obtained by the calculation of more than 2000 data points, respectively.
4. The Arrhenius-type model is not very qualified to accurately represent the flow behavior of annealed TA31, only using one set of parameters over the range of both α and α + β. However, the modified JC model describes the characters of annealed TA31 from dynamic recrystallization to dynamic recovery, leading to the change of the curves’ shape.
5. The modified JC model does not only have a simple mathematical expression, but also has the ability to predict the stress of TA31 accurately under a set of model material parameters. It will reduce the cumbersome and complex programming when this model is implemented into the finite element software using the subroutine.

## Figures and Tables

**Table 1:** The main chemical composition of TA31 [27].

Element	Ti	Al	Nb	Mo	Zr	Fe	C	H	O	N
wt.%	Bal.	6.08	2.94	1.02	2.02	0.038	0.005	0.001	0.071	0.003

**Table 2:** The material parameters of TA31 for the Arrhenius-type model by regression method.

i	α (MPa^−1^)	*n*	ln*A* (s^−1^)	*Q* (J/mol)
0	0.02357	3.513363	82.93772	847,968.6
1	−0.02839	0.269352	−178.989	−1,786,072
2	0.149064	−6.66775	681.1676	6,839,654
3	−0.28922	12.23534	−1547.92	−15,672,804
4	0.263723	−8.70912	1732.279	17,655,308
5	−0.10499	2.590169	−760.466	−7,789,986

**Table 3:** The initial material parameters of TA31 for the modified JC model.

*E*_0_			*E*_1_			*E*_2_			*E*_3_			*E*_4_			*E*_5_		
0.05			0.01			0.01			0.01			0.01			0.01		
***K*_1_**		***K*_2_**			***K*_3_**			***K*_4_**		***T*_m_**			***T*_r_**			${\dot{\varepsilon }}_{0}$	
40		−0.001			−0.1			0.001		1600			850			0.01	
***F*_0_**	***F*_1_**			***F*_2_**			***F*_3_**		***F*_4_**		***F*_5_**			***F*_6_**			***F*_7_**
1	1			1			1		1		1			1			1

**Table 4:** The material parameters of TA31 for Arrhenius-type model by optimization method.

i	α (MPa^−1^)	*n*	*LnA* (*s*^−1^)	*Q* (J/mol)
0	0.001837	6.065099	99.73633	847,968.9
1	−0.00211	−2.61215	−191.982	−17,86,072
2	0.023596	2.722948	690.1866	6,839,654
3	−0.06051	−1.62176	−1552.48	−15,672,804
4	0.07645	−8.32858	1723.548	176,55,308
5	−0.03442	11.15299	−753.387	−7,789,986

**Table 5:** The material parameters of TA31 for modified JC model by optimization method.

*E*_0_			*E*_1_			*E*_2_			*E*_3_			*E*_4_			*E*_5_		
0.3784			0.1446			0.2165			0.1648			−0.0165			0.0040		
***K*_1_**		***K*_2_**			***K*_3_**			***K*_4_**		***T_m_***			***T_r_***			${\dot{\varepsilon }}_{0}$	
21.8063		−0.2743			−1.00			0.0017		1600			850			0.01	
***F*_0_**	***F*_1_**			***F*_2_**			***F*_3_**		***F*_4_**		***F*_5_**			***F*_6_**			***F*_7_**
−15.1344	3.0181			−1.11674			42.8451		0.2102		0.0128			50.00			−1.1794

## Data Availability

The raw/processed data required to reproduce these findings cannot be shared at this time as the data also form part of an ongoing study.
